# Commentary on the “A multidisciplinary opioid-reduction pathway for robotic prostatectomy: outcomes at year one”

**DOI:** 10.1186/s13741-024-00392-w

**Published:** 2024-07-11

**Authors:** Binbin Zhu, Angyang Cao, Yijun Chen

**Affiliations:** 1 Anesthesiology Department, The First Affiliated Hospital of Ningbo University, Ningbo, China; 2 Health Science Center, Ningbo University, Ningbo, China

## Abstract

**Background:**

Opioid-sparing multimodal analgesia is increasingly emphasized for postoperative pain management. This commentary discusses a study by Manning et al. on an opioid reduction pathway for robotic prostatectomy.

**Methods:**

We reviewed the Manning et al. study, which implemented a multidisciplinary opioid reduction pathway and compared outcomes before and after pathway implementation. Outcomes included opioid use, pain scores, antiemetic use, length of stay, and readmissions.

**Results:**

The study found reduced opioid consumption, lower antiemetic use, shorter length of stay, and similar pain scores after pathway implementation. However, the pre-post-study design has limitations in attributing causality to the pathway itself. Key confounders were not fully accounted for. The clinical significance of the small reduction in length of stay is also questionable.

**Conclusions:**

This commentary highlights important limitations of the Manning et al. study, including the retrospective design, potential confounding factors, small effect size, and lack of long-term outcomes. While the study provides early evidence for a multidisciplinary opioid reduction approach, further rigorous prospective research is needed to confirm the observed benefits and long-term impacts. Additional focus on direct opioid consumption, equivalent analgesia assessment, and clinically meaningful outcomes is warranted.

Dear Editor,

We read the manuscript “A multidisciplinary opioid-reduction pathway for robotic prostatectomy: outcomes at year one” with great interest (Manning et al. 2023). This is an important topic and the authors’ effort to reduce opioid use through a multidisciplinary approach is commendable. However, I have some concerns regarding the study methodology and conclusions:

This is a retrospective study comparing outcomes before and after the implementation of the opioid reduction pathway. However, there may be other confounding factors that changed over this period that could also explain the observed differences. There is currently no global standardization of the definition of low opioid use (Wick et al. 2017). Table 1 in the accompanying document to Manning’s paper describes the “opioid-free pathway.” However, the percentage of patients taking acetaminophen, ibuprofen, celecoxib, ketamine, lidocaine, dexmedetomidine, ketorolac, hydromorphone, morphine, fentanyl, ondansetron, dexamethasone, haloperidol, diphenhydramine, esmolol, desflurane, metoclopramide, promethazine, gabapentin, naproxen, or oxycodone was not described. More details can be listed in table form. Meanwhile, when multiple interventions are implemented simultaneously, as with their opioid reduction pathways, it is difficult to establish a clear cause-and-effect relationship between any single component and the observed outcomes. There are a number of potential confounding factors at play, such as administrative pressure, patient and provider attitudes toward opioids, and the Hawthorne effect, which may help reduce postoperative opioid use. The fact that path adherence is actively monitored and targets are set only adds to concerns about the Hawthorne effect affecting outcomes. A prospective study or randomized trial with consistent fixed therapy would provide stronger evidence for the impact of the pathway itself.

The application of gabapentin in access after operation is particularly noteworthy. Gabapentin is known to cause sedation, especially in the elderly or in patients with impaired kidney function. If this sedation resulted in a reduction in opioid demand, then it may have artificially reduced the observed opioid consumption in the post-implementation group, without necessarily reflecting adequate pain control. The authors did not report sedation scores or any other measures of cognitive function. This is an important oversight because it makes it impossible to understand whether the opioid-saving effect is really due to the effectiveness of a multimodal analgesic intervention or may be confused with sedation. In addition, gabapentin is an analgesic, not a direct analgesic. If the goal is to reduce opioids, then including sedative non-opioids like gabapentin seems counterintuitive and potentially problematic.

The authors conclude that the pathway resulted in reduced opioid use and shorter length of stay, while maintaining similar analgesia. However, patient-reported pain scores may not fully capture analgesia. The pain scores in both groups may be low due to less invasive robotic prostatectomy. A larger patient population is necessary to detect significant differences in pain control. It is important to consider if pain scores reflect resting pain or motor pain. If opiate-free patients struggle with dynamic pain, it suggests that reported measures may not detect suboptimal pain management. Assessing pain scores during rest and activity will offer a more thorough evaluation of analgesic effects. Patients without opioids may struggle with walking due to insufficient pain management. Reduced opioid use affecting a patient’s ability to promote recovery may have unreported consequences. Direct measurement of opioid consumption would better assess if analgesia was truly equivalent between groups.

From the patient’s perspective, the clinical significance of the reduced length of stay is unclear. The absolute difference in means of 0.2 days for a hospital stay is quite small. But when it comes to hospitals, even a seemingly small difference in length of stay could have meaningful implications for healthcare systems, particularly those with constrained hospital capacity and limited turnover of beds. In settings where bed availability is tight, shaving even a few hours off a patient’s length of stay can have a tangible impact on a hospital’s ability to efficiently manage patient flow and accommodate new admissions. Furthermore, long-term follow-up on opioid use and pain outcomes is lacking. The benefits of reduced short-term opioid exposure are uncertain without assessing longer-term impacts.

The participant groups before and after pathway implementation are not completely matched. There are small but statistically significant differences in BMI, BSA, and smoking status. Better matching on baseline characteristics would strengthen the analysis.

The decline in post-anesthesia care unit (PACU) residency duration presents an intriguing scenario that could have various contributing factors. The use of desflurane reduces PACU time compared to other volatile anesthetics such as sevoflurane and isoflurane. The authors mention that desflurane is specifically recommended for patients with BMI ≥ 30 as part of an opioid reduction pathway. However, detailed information on inhaled anesthetic use between the two groups was not provided. This is an important omission, as differences in PACU duration may be partly or even primarily driven by inhalant selection, not just opioid intervention. Without understanding the distribution of volatile anesthetic use across the cohort, it is difficult to attribute the reduction in PACU residence time to a multidisciplinary approach alone.

In summary, this study provides early evidence to support a multidisciplinary opioid reduction approach, but the results must be interpreted cautiously given the limitations. Additional rigorous prospective research is needed to confirm the observed benefits and long-term impacts. We hope the authors will consider these suggestions to strengthen their important work in this area.

## Data Availability

Not applicable.
